# Alfuzosin for chronic pelvic pain syndrome: Another placebo?

**Published:** 2009

**Authors:** T. J. Nirmal, J. C. Singh

**Affiliations:** Department of Urology, Christian Medical College, Vellore, India E-mail: chandrasingh@cmcvellore.ac.in

The most common urologic diagnosis made in men younger than 50 years is prostatitis.[2] Till date, a multitude of studies involving various therapeutic agents have been conducted.[2] Lack of a clear etiology is an important limitation in identifying an appropriate treatment strategy. Though the efficacy of the alpha-adrenergic blocker alfuzosin in treating the symptoms of chronic prostatitis/chronic pelvic pain syndrome (CP/CPPS) has been studied before, results have not been consistent.[3,4] Most of the other trials that demonstrated a favorable outcome were underpowered. The results of a meta-analysis of alpha-adrenergic antagonists in men with CP/CPPS were that there was a significant reduction in total NIH-CPSI score and IPSS. But the authors had emphasized that the duration of treatment needs to be at least three months to see the effect.[5] This trial[1] and that of Alexander *et al*.[4] were adequately powered but both concluded that alfuzosin is not beneficial in CP/CPPS. One of the reasons attributed to the lack of benefit in the trial by Alexander *et al*.[4] was that patients were not alpha-blocker naive. But, though only those who had never been treated with alpha-blockers were included in this study,[1] alfuzosin was found to be ineffective. The impact of alpha-blockers on men with concomitant outlet obstruction was not studied separately in this trial. Furthermore, as the duration of treatment was only six weeks the possibility of benefit with longer duration in alpha-blocker naive patients remains to be studied. One of the known adverse effects of alpha-blockers is retrograde ejaculation. Though the incidence is lesser with alfuzosin compared to tamsulosin, the finding that there was significant improvement in scores for ejaculation following therapy with alfuzosin in this trial comes as a surprise. Whether the improvement was actually in the pain during ejaculation or ejaculation *per se* is not clear. Other therapeutic options being explored for CP/CPPS include tricyclics, anticonvulsants, physical therapy, and myofascial or trigger point release.[2] The results in favor of these interventions have not been consistent. By this trial, we know that short-term alpha-blocker monotherapy is not useful for alpha-blocker naive patients. But multimodal therapy needs to be explored addressing various aspects of CP/CPPS, including adequate pain relief, improvement in voiding symptoms, and quality-of-life issues.
